# Death and Dying in Plastic and Pictorial Art

**Published:** 1910-12-03

**Authors:** 


### SPECIAL ARTICLE.

### DEATH AND DYING IN PLACTIC AND PICTORIAL ART.*

"Fear death—the Arch Fear in a visible form?" asks Browning in the often-quoted "Prospice," and it is a question that every reasonable being—reasonable not merely in the sense that our quibbling law comprehends in an indictment for murder—has at one time or other asked himself. The answer must necessarily depend on individual sentiment and character. The heroic *insuper et caro mea requiescet in pace, quoniam a dextris est mihi ne commovear* of the Vulgate, Browning's enthusiastic "No," and Dante's fierce *Taci, maledetto lupo!* are variants, after all, of the same philosophy that finds in death not the end, but, as Longfellow wrote, the transition to a higher state. Whether that feeling rests on the Αὐτὸς ἔφη or on the primal instinct of those that

Errare atque viam palantes quaerere vitæ

is little to the purpose. The concrete fact remains that the majority of mankind face death with a remarkable stoicism. Medical men who have devoted some attention and observation to the details of deathbed scenes agree that the popular idea of the mental agony that precedes dissolution is luridly overdrawn. Nevertheless popular fancies, however at variance with established facts, have their place in art, and it is an interesting discussion how widely this particular assumption of inherent fear has influenced artists in depicting in pencil, colour, or plastic work, incidents connected with the various aspects of dying.

In this study of the question Dr. Weber has exploited the treasures of his own collection of coins and medals to furnish some guiding lines on which an estimate of such development may be based. In an elucidative preface he declares that his attempt is "not intended to be an essay on the iconography of death, but on the mental attitudes towards the idea of death, and the various ways in which it has, or may be supposed to have, affected the living individual—his mental and physical state and especially the direction and force of his action—as illustrated by minor works of art." This limitation is necessary in view of the vast amount of material that lies at hand, ready for the energetic investigator who hereafter may turn his attention to the whole subject. Necessarily within the limits which the author has set himself there are enough examples and illustrations to fall back upon, and it may, in the first place, be wondered why, after a scholarly introduction that gives promise of such brilliant performance, the author has confined himself strictly to his marginal scheme. The philosophic discussion of death and dying presents so much that is attractive that it is perhaps well, however, that these pales have not been overstepped. The subject is so vast that it is certain Dr. Weber has not exhausted its capitular classification with the eighteen divisions into which he has arranged it. We have here (i) the simple *memento mori* idea; (ii) death as the threshold of a future existence; (iii) posthumous fame; (iv) death as the end of pain and misery; (v) as vengeance or atonement; (vi) as the emblem of war or effort; (vii) as emblem of destruction and ruin; (viii) as the common leveller; (ix) its scientific aspect; (x) the prevention of death; (xi) death as self-sacrifice; (xii) the epicurean attitude; (xiii) mindfulness of death as a pure philosophy; (xiv) the same as an incentive to personal service; (xv) death as the crown of life; (xvi) predestination; (xvii) the pessimistic attitude; and (xviii) grief for the departed. The list might be extended and one could find texts, historical and literary, to illustrate it. Where, for instance, in the above classification are we to place the conception of death, not as the crown of life, but as the index of what life was worth—the conception that Grillparzer embodies in his well-known lines:—

Was etwa noch übrig geblieben

Ist erst nach dem Tode wahr!

and to which de Genestet gave such poignant expression in his poem on the child dying in its mother's arms? Where the idea of death as a purification which lingers in the Heitsi Kabib legend of the Khoi Khoi? Where, finally, the feeling that death itself may be annihilated, that cryptic supposition of a nirvana which, as Nordau has somewhere pointed out, occidentals cannot grasp with the supreme totality that is attained by eastern nations? Possibly—we speak with great diffidence, for the author is by no means clear as to his conception of Epicurean philosophy—Dr. Weber would refer most of these to the twelfth, fifteenth, and seventeenth divisions. He quotes Symonds' opinion of the finest Latin poem extant with obvious satisfaction: we can only refer him to "De Rerum Natura" (and even to Motee's carping criticism on it) for a proof that Epicureanism as understood by Lucretius was not wholly pessimistic, Numenius and Eusebius' comments notwithstanding.

But this, after all, is a side issue: the fact remains that Dr. Weber has handled his huge subject and the mass of material at hand with commendable conciseness and clarity. He has drawn freely upon a large collection of interesting medals, coins, tokens, plaques, cameos, gems, and similar curiosities, and described examples with a detail that shows how much a labour of love the task has been to him. It is obviously impossible to deal with these in the limits of a brief review. The majority appear to concern themselves largely with the simple *memento mori* idea. Such, for example, are the iron memorial medal of Blumenbach, the Joachimsthaler Pestmedaille, and many others, of which he describes several dozens. The section dealing with prints and pictures (introduction) is much less full, but here again the field is so vast that it is surely impossible to be catholic. We notice, however, that the author

* *Aspects of Death in Art.* By F. Parkes Weber, M.A., M.D., F.R.C.P., F.S.A. London: T. Fisher Unwin. 5s. net.)

mentions Burgkmair's print of death strangling the young lover, but omits the even more interesting—and, we believe, earlier—example of this type of work in which death is represented as throwing spears or shooting arrows at mankind (see, for instance, the Totentanz von Meister H.W. 1482). Chodowieki's interesting print at Graz, a replica of which is in the Berlin Museum, representing death and a doctor, might also have been mentioned. While attention is paid to Blake's fantastic designs, the bizarre fantasies of Wiertz, and the more restrained work of Watts is not remarked upon: Boecklin's world-famous genre pictures are included. Where such embarrassing potentialities for descriptive purposes exist it is perhaps ungenerous to express a wish that the author has not included other things. But we look forward to a fuller, wider, and more comprehensive treatise from Dr. Weber's pen on the same subject in the future, and we trust that he will not overlook the many interesting examples of sculpture in which death and dying in their various forms are represented in some way. Also we would put in a plea for the less well-known wood paintings such as the comparatively unstudied Eetbordges of the late sixteenth century, examples of which are in the Ryks Museum at Amsterdam, and of which we have recently seen one in the private collection of a distinguished medical *confrère* in London. Although these do not possibly express the *memento mori* idea in its full significance—and it is hardly to be wondered at when one considers that these works of art were meant for table decoration—they yet deserve study in this connection, and would repay some attention from so enthusiastic and pains-taking an observer as the author.

Much of this book is of great interest to medical men. The subject is fascinating, not merely in its morbidness, but through its solid human interest. Most of us would willingly agree with Rabbi Hillel that death presents various aspects only to the unwise. But we are all foolish at some periods of our existence: there is a diurnal variation in the degrees registered by our temperamental barometer, and not the least interesting speculation centres itself around the question in how far our fear of death, our apprehension of the unknown that Epictetus called the inevitable crisis, is influenced by these oscillations. There is nothing detrimental in the study of death, and when it is combined, as Dr. Weber combines it, with an investigation into the development of certain definite ideas and their elaboration in art, it becomes absorbingly enthralling and indubitably instructive. To those who wish to pursue the subject further we cordially recommend this book. It is brief, clear, admirably expressive in its language, well illustrated, and in every way suitable to be awarded a permanent place on the shelf where we conserve our army of friends in the book world.

